# Why Does the Biosphere Interact with Earth’s Climate, and How Do the Two-Way Interactions Work?

**DOI:** 10.3390/biology13121040

**Published:** 2024-12-12

**Authors:** Shoichiro Nakamoto

**Affiliations:** Ryukyu Perimeter Institute, 321-16 Biimata, Nago City 905-0005, Okinawa, Japan; nakamotoocean@aol.com; Tel.: +81-0980-53-4177

**Keywords:** oceanic radiative transfer, ocean physics modulated by phytoplankton, non-equilibrium, complexity, entropy production, self-organization, food web network, noise-induced irreversible jump, information template for heredity, bio-geophysical climatic interaction

## Abstract

With the use of matter (carbon dioxide, nutrients, and water) and solar energy, phytoplankton produce oxygen and carbohydrates, which are transported to predators through the oceanic food web hierarchy. From the viewpoint of irreversible processes of non-equilibrium thermodynamics, oceanic photosynthesis gives a mechanistic picture of living things characterized by double sets of self-organizations supported by flows of energy and entropy discarded into the ocean environment. This produces biological, ocean circulation, and climate interactions.

## Simple Summary

Phytoplankton are like workers in the biospheric food factory. They photosynthesize carbon dioxide, nutrients, water, and the Sun’s light to produce carbohydrates, and they release oxygen and heat into the ocean. From a non-equilibrium thermodynamic mechanistic view, photosynthesis accounts for life on Earth. The two-way interactions of phytoplankton with geophysical fluids lead to a network relationship that connects small-scale phenomena to those on larger scales, which has climate implications.

## 1. Introduction

The term biosphere was first introduced by an Austrian geologist, Edward Suess, toward the end of a book on Alpine geology, and again introduced by a mineralogist, Vlagimir Ianovich Vemadosky of the Soviet Union, in 1926. They concluded that the biosphere is the sum total of all living beings that interact to form complex biological networks. The population of the biosphere changes very rapidly (some micro-organisms live less than a day even the oldest living organisms the bristlecone pines live less than five thousand years) compared to the age of the Earth. A National Research Foundation-sponsored report in 1983 stated that “Improved understanding of the biosphere and its relation to the other great domains of the earth has a special urgency.” [1].

The climate can be described as the long-term effect of the Sun’s radiation on the rotating Earth‘s varied surface and atmosphere. In 2000, the American Meteorological Society published the *Glossary of Meteorology*, in which climate is defined as “the slowly varying aspects of the atmosphere-hydrosphere-land surface system, characterized in terms of suitable averages of the climate system over periods of months or more, taking into consideration of the variability in terms of these averaged quantities” [2]. A simple long-term summary of weather changes is not a true picture of climate. To obtain this requires an analysis of daily, monthly, yearly, and decadal patterns. Investigations of climate change over geological time require the tools and methods of geological research [3]. Spatial scales are additional information needed for the definition of climate [4]. In 1998, Beniston wrote that “knowledge of two-way interactions between biosphere and atmosphere are poorly known for their effects to adequately included in a present-day climate modeling” [5]. Understanding the interaction between the biosphere and climate requires broad ranges in both spatial and temporal scales in physical, chemical, geological, and biological mixtures. This is why climate study is considered a science of complexity systems [6,7,8].

It is worth recalling that the “Biological influence on Climate Physics” was conceived during the “Surf-side ClimateWorkshop on Climate Forcing in Oceanic Ecosystems” held in 2001 [9]. At the workshop, a challenging hypothesis, “Does varying phytoplankton biomass in the ocean affect oceanic radiation transfer process, and thus, modifcation of the ocean circulation and the climate state?” was posed.

To address this issue, my report is organized as follows. In Section 2, I examine this from the viewpoint of irreversible non-equilibrium thermodynamics. The phenomenology of photosynthesis is discussed in terms of living beings on Earth, aiming to obtain a generalized picture of “Life”. Section 3 addresses the observational and theoretical work on oceanic photosynthesis and its interaction with the ocean environment. The report concludes with a discussion of future directions and remarks in Section 4. These Sections weave together six scientific themes: non-equilibrium thermodynamics, complex systems, modeling, local equilibrium for the Navier–Stokes equations, transport in diffusive systems, and thought experiments. Background on these themes is provided in Appendix A, Appendix B, Appendix C, Appendix D, Appendix E and Appendix F.

## 2. Why Does Phytoplankton Interact with Its Environment?

In a little book entitled “What is Life”, Erwin Schrodinger set out in 1944 to explain why biological organisms seemed so mysterious from the classical physics viewpoint. At that time, living organisms were viewed as nothing more than elaborate machines with microscopic parts. Schrodinger argued that the stable transition of genetic information in discrete bits implied a quantum mechanical process. The role of cogwheels of macroscopic clockwork may have inspired Schrodinger to pose the proposition of information bits in life. This beautiful little book was immensely influential in the early days of molecular biology. In 1952, a chemical recipe for life led to the idea that we could mix up the right stuff in a test tube and make life in a laboratory, which led to the famous Miller–Urey experiment. But this recipe turned out to be a blind alley. Since then, physicists have long been puzzled over life because the simple application of the second law of thermodynamics is applicable only to closed systems, and entropy estimates in the traditional chemical reaction formalism of photosynthesis forbid the progress of any chemical reaction process. Since then, there has been considerable interest in this area. In 1980, David Bohm wrote, “the seed contains information, in the form of DNA, and this information directs it to form a corresponding plant” [10] (p. 194). Carl F. Weizsacker, independently, wrote, “The hard core of thermodynamics is the Second Law with entropy, i.e., information as the distinguished quantity”(p. 300) [11]. In 2000, Yoshimi Ohta and Kazumichi Nakagawa of Kobe University conducted a laboratory experiment to measure the rate of energy conversion by phytoplankton from incoming visible light to heat, which is discarded to the environment. They reported that the conversion rate was 82% in cell growing periods and 92% in non-cell growth [12]. In October 2002, the US Space Agency-supported workshop “Quantum Aspects of Life” was held in California. At this workshop, Paul Davies argued that all living organisms are information processors: they store a genetic database and replicate it, with occasional errors, providing the basis for natural selection [13]. At the same workshop, Alexandra Castro, Francesca Olsen, Chiu Fan Lee, and Neil Johnson gave a talk entitled “Ultra--fast Quantum Dynamics in Photosynthesis” [14]. They argued that after a photon is absorbed by a light-harvesting antenna, the central step in photosynthesis is excitation transfer to a molecular complex that serves as a reaction center (where the excitation is trapped), thereby allowing charge separation to take place.

The excitation transfer of an electron is a dark reaction that takes only a few hundred picoseconds and is performed as a self-catalytic chemical reaction. It is worth remarking that the process of self-catalysis does not explicitly appear in the chemical reaction formalism, because the chemical reactions appear to a macroscopic observer to be like some sort of invisible hand of God. However, the process of self-catalysis is extremely important in order to understand the whole process of photosynthesis from a thermodynamic viewpoint.

Photosynthesis, in the realm of equilibrium thermodynamics, leads to inconsistencies because the macroscopic picture of photosynthesis is a manifestation of a quantum mechanical process. It is worth recalling that Carl F. Weizsacker wrote that “Modern physicists must ask how can traditional physics be interpreted in the light of Quantum theory” (line 16–19, p. 144) [15]. Schrodinger pointed out entropy production by living things in the book “What is Life?”. Thus, the entropy of sunlight in the process of absorbing light quanta in photosynthesis illustrates a new look at “Life”. This is because the whole process of photosynthesis encompasses multi-agent interactions spanning from a quantum mechanical scale of 10^−15^ s to a physiological scale of 10^5^ s.

Since phytoplankton can influence ocean physics, as shown by optical physicists and biological oceanographers (see Section 3), the effect of phytoplankton connects to geophysical scales and eventually to climatic disturbances. Here, mathematical nonlinearity plays a key role in both chemical reaction processes and geophysical fluid dynamics.

The analogue of mathematical nonlinearity in chemical reaction formalism was discussed by Prigogine and Stengere, who demonstrated a “Thought Experiment” to obtain a mechanistic model of photosynthesis that characterizes the birth of a new state. See Appendix F for a discussion of this concept. They argued that, because the control parameters regulating irreversible processes, i.e., growth, mutation, or reproduction in biological problems, cannot be considered as constants, an external forcing in the nonliving environment triggers the birth of a new macroscopic type of behavior inside the system [16]. These processes are intrinsic transformations to more complex systems. In nonlinear mathematical systems, these are well known as “hysteresis phenomenon” irreversible jump processes induced by external noise in real-world complexity systems was coined as a co-existence of multiple equivalent stable states by Prigogine and Stenger.

At many functional levels, biological processes are characterized by irreversible changes or macroscopic behavioral changes over time, which may be interpreted as an irreversible transformation from one structural state to another. Such phenomena are routinely characterized by episodes of increasing organization and complexity. Prigogine and Stengere stated that such processes are representative of non-equilibrium thermodynamics by setting the division of systems into those whose behavior is controlled by boundary conditions for living things influenced by the external environmnt, and those controlled by their initial conditions inherited in the system. The former are interpreted as the transport of energy and matter for supporting chemical reactions that satisfy the causality principle of physics. The latter are considered an information template that controls heredity and reproduction, which Bohm and Weiszacker might have expected. Brooks and Wiley further expanded these two aspects as follows: the development of multi-cellular organisms must be caused both by the external state of the environment and by temporal interaction in which the organism develops [17].

The genetic program of living organisms has drawn attention from theoretical biologists. For instance, in 1998, at the Fundamental Physics Lab. of Kyoto University, Masatoshi Murase gave a lecture entitled “LIFE AS HITORY”. He argued that the characteristics of life (i.e., reproduction, evolution, and mutation) that achieve “adaptive power” to cope with unexpected environmental changes are historically inherited ever since a birth of life on earth [18]. It is interesting to recall that Murase’s wording of “HISTORY” resembles a “biological hysteresis” inherited on evolution time scales. Neil Johnson pointed out that such an adaptive ability was an essential element for the definition of a “Complexity System”. Johnson interpreted such an adaptive power of evolution as “feedback” between the living beings and the varying physical environment, i.e., living things can continue their activities by discarding entropy to the Earth environment, and eventually that entropy is discarded to the colder outer universe in the form of the infrared spectrum, which climate scientists call OLR (outgoing long-wave radiation).

External random physical forcing may trigger a new type of behavior in the interior biological system, involving much more complex reaction schemes. Prigogine and Stengere coined this as “a birth of a macroscopic system”. For example, some macroscopic rhythmic senses of electric currents detected from living plants indicate a “fractional Brownian motion” that may be a macroscopic remnant of “Life” [19,20,21].

Why does phytoplankton need to interact with its environment? The answer is that “through exchanging energy and matter with external environment, phytoplankton can execute the process of photosynthesis, which is irreversible and produces entropy”. Why does the traditional chemical reaction formalism not include this entropy production? Perhaps it is because some self-catalytic processes in the dark reaction of photosynthesis are hidden from macroscopic observers. In order to identify the self-catalytic chemical reactions in the process of photosynthesis, Progogine and Stengere employed a “Thought Experiment”, a concept often used by theoretical physicists.

A “Thought Experiment” is also needed in order to interpret the laboratory experiment by Ohta and Nakagawa at Kobe University. This forms the connection between logical positivism and a new paradigm. That is, the result of the lab experiment by Ohta and Nakagawa was based on the macroscopic detection of sunlight and water temperatures, which can be interpreted by the language of quantum mechanics. I note that a quantum mechanical experiment of photosynthesis was proposed by Alexander Castro and her associates in London in 2003.

It is worth noting that logical positivism in scientific reductionism is a strong tool for setting up mechanistic models. However, that is not the whole story of complexity systems with interacting multi-agents. In such a case, a “Thought Experiment” bridges the gap between “logistical positivism in classical reductionism” and the “ontological existence of multi-agents interaction processes in the real world”. This is “a leap in human epistemology” as taught in the history of science courses. Notice that Weiszacker emphasized “the leap in human epistemology” by using such a wording in “Eine Revolution in der Physik” (line 14 of p. 93) [15].

Employing the terminology introduced by Nicolis and Prigogine, the conclusion is as follows: “Photosynthesis executes microscopic multi-agents involving self-organization that is observed as spatial patterns or temporal rhythms at macroscopic scales” (p. 8) [22]. The process requires the input of chemical and other energy sources needed to maintain the living state, i.e., “Life”, on Earth.

To summarize, phytoplankton are two-fold self-organizations: a macroscopic organization and a microscopic self-catalytic organization. Both are driven by the causality principle of physics. The macroscopic organization is possible when flows of energy and matter are allowed to support bio-chemical reactions to produce a self-organized structure inside the system. This process is commonly called a “dissipative structure” in non-equilibrium thermodynamics (see Appendix A). The microscopic self-organization, i.e., the electron excitation transfer process of phytoplankton, is hidden from macroscopic observers.

## 3. How Do the Two-Way Interactions Work in the Ocean?

Phytoplankton are primary producers through photosynthesis. This produces low-entropy products, which are transported to predators that support the biosphere [23]. How can such a production of lower-entropy matter be achieved by phytoplankton alone? In this section, I review the published oceanic photosynthesis research from the viewpoint of non-equilibrium thermodynamics.

Phytoplankton are like workers in a biosphere food factory. They use materials such as carbon dioxide, nutrients, water, and sunlight energy to produce oxygen, carbohydrates, entropy, and heat. The latter two are discarded into the ocean environment. This production requires three basic elements: raw materials, laboring facilities, i.e., phytoplankton, and labor to breathe “life” into products. These three basic elements are the main ingredients for photosynthesis. During the course of evolution, plants in the aquatic environment have one major advantage over their terrestrial cousins, namely, an abundant water supply. However, aquatic water both absorbs and scatters incoming sunlight. Thus, phytoplankton are responsible for determining the optical properties of sea water.

The rate of photosynthesis depends on the rate of capture of light quanta in the blue-to-red frequency range. The critical factor is the number of available photons, rather than their total energy. Thus, a photon of wavelength 400 nm, if absorbed by chlorophyll, induces the same chemical change as does a less energetic photon of wavelength 700 nm [24]. Laboratory measurements by Ohta and Nakagawa showed that the rate of conversion from sunlight energy into heat is extremely large. This means that only a part of the photon energy appears to go to photosynthesis and energy in the form of heat, the latter being emitted into the ocean environment. The process of conversion from sunlight energy and intake energy to entropy discarded as heat is a macroscopic example of a photosynthesis self-catalytic reaction. This fact has not appeared explicitly in the traditional macroscopic formalism of the chemical reaction of photosynthesis.

Natural waters, both fresh and saline, are “a witch’s brew of dissolved and particulate matters” according to Mobley, because the availability of light is the limiting factor for primary production by aquatic ecosystems [25]. For phytoplankton and microphytes in lakes, rivers, and coastal waters, the intensity and spectral quality of the light varies with depth. The vertical profile of the radiation heating rate was estimated by Siegel and Dickey [26]. Simonot et al. reported that the seasonal cycle of phytoplankton has a significant impact on Sea Surface Temperature simulations [27]. Ever since the pioneering theoretical proof of the oceanic radiative transfer process by Preisendorfer and Mobley, research activities in radiative transfer theory and optical oceanography have influenced biologists, chemists, physicists, ecologists, and oceanographers in different countries [28,29,30]. In 1989, the American Society of Limnology and Oceanography published an encyclopedic special issue of ocean optics and oceanic photosynthesis in “Journal of Limnology and Oceanography”, which include “Field measurements” and “Individual particle assessment”. In addition to this special issue, Curtis Mobley’s monograph “Light and Water” summarizes modern observational and theoretical results in radiative transfer in the ocean [31,32,33,34].

Thermodynamic interactions of biology and physics in the interior ocean are not negligible, as pointed out by ecologists [35]. That is, some portion of solar energy converted into chemical energy is stored in phytoplankton. This is then transferred to zooplankton and other sea animals under a food web hierarchy network. Thus, a phenomenological description of the oceanic food web is needed to illustrate the flows of energy and matter in the ocean interior.

There is another important interaction between ocean physics and phytoplankton. At the dawn of remote sensing technology, biological oceanographers pointed out a phenomenon of a significantly higher chlorophyll concentration in the Sulu Sea, between Borneo and the Philippines, than in adjacent seas. The cause of such high chlorophyll concentrations is believed to be supplies of nutrients from the seabed, mostly by turbulent mixing from atmospheric forcing by the wind, precipitation, and sunlight penetration into the ocean. In the Sulu Sea, internal gravity waves, generated by interactions among wind, pressure fluctuations, and flows over the bottom topography, may also be a major cause of water mixing in the vertical direction.

Mean and eddy flows over the bottom topography generate internal lee waves, while tidal flows over the topography generate internal tides, developing to internal gravity waves. Thus, atmospheric disturbances generate internal gravity waves in the ocean, which enhance the vertical mixing of sea water, leading to a supply of nutrient-rich water throughout the water column. In turn, this supports phytoplankton blooming and, ultimately, modification of the meridional heat flux in the ocean. This illustrates an important and heretofore unknown interaction between small-scale geophysical fluid disturbances and oceanic organisms that connects small-scale internal gravity waves and enhanced phytoplankton blooming to the modulation of global-scale ocean circulation dynamics [36,37,38,39,40,41,42,43]. In short, solar energy is both absorbed and scattered by phytoplankton, micro-algae, related pigments, detritus, and dissolved organic matter. The resultant energy radiates downward into the ocean interior by geophysical and biological processes.

This fact indicates that life-cycle-related entropy discarded by the oceanic biosphere, i.e., both phytoplankton and oceanic organisms such as zooplankton, micro-animals, and sea animals, including the benthic communities, are an important heat sink in interior ocean waters. This then influences long-term climatic implications in global-scale ocean circulation. It is emphasized that small-scale geophysical flows, such as internal gravity waves in shallow seas, cause biophysical multi-agent interactions between phytoplankton and global-scale ocean circulation physics. Simply put, phytoplankton blooming in the shallow sea is caused by the vertical mixing of sea water due to internal gravity waves, leading to modifications of ocean density that can affect basin-scale oceanic meridional heat transport [43].

## 4. Discussion, Future Directions, and Concluding Remarks

The question of “why and how do the two-way interactions between phytoplankton and the Earth environment work?” motivated me to look at oceanic photosynthesis from the point of view of “non-equilibrium irreversible thermodynamics”. This is the basis of “Life on Earth”. By “strictly non-equilibrium”, I mean photosynthesis in an open system that allows flows of energy and matter to drive intrinsic chemical reactions of phytoplankton. This is a typical example of physics–chemistry–biology connecting multi-agent network schemes implemented by the complexity system that is the “Earth system”. Notice that weak non-equilibrium thermodynamics allows some linear transport processes that approach a time-symmetrical state, often called a “local equilibrium state” in the classical equilibrium setting of physics models. From the non-equilibrium thermodynamic view, I looked at photosynthesis as a macroscopic phenomenon characterized by metabolism, entropy production, growth, and the birth of multi-equivalent states.

However, these characteristics are not related to an “information processor” that Schrodinger, Bohm, and Weiszacker might have expected. This means that the characteristics of “life” are controlled by the laws of physics and chemistry and, thus, cannot be the answer to Schrodinger and Bohm’s questions. Schrodinger raised issues about not only entropy production but also genetic information. Entropy production obeys the fundamental principle of physics that controls phenomenology for the living, as well as for the inanimate.

Phytoplankton photosynthesis is one aspect of “Life”. It is a wonderful example of a self-organizing complexity system controlled by a mechanistic model that obeys the causality principle. It also illustrates a two-fold macroscopic self-organization regulated by the principle of physical causality, and a microscopic self-organization controlled by hidden self-catalytic chemical reactions. The former is directly driven by flows of energy and matter to produce self-organized structures. The latter involves autonomous self-organization by self-catalytic chemical reactions, that is, by quantum mechanical manifestation. Note also that mathematical bifurcation is completely controlled by the causality of physics laws, but the action of choosing the future state by phytoplankton is stochastic because it is controlled by an inherited information template with accidental errors due to noise [44].

In 2003, Paul Davies asked where is the “information processor”, an essential player in genetic transformations. Recall that the birth of multiple equivalent states is consistent with the bifurcation phenomenon of nonlinear mathematical branching processes without external noise. In the case of external noise forcing, stochastic perturbations of choosing branches dominate the process of physical determinism, which is controlled by the principle of minimum energy and maximum entropy, itself controlled by the causality principle of physics.

Ecologists interested in the diversity and sustainability of life pay attention to food web networks in the ocean. This is because flows of energy and matter in the ocean interior are controlled by the biological activity of sea animals, as well as ocean circulation. However, the oceanic food web scheme is poorly known, and the topic of the oceanic food web has drawn much attention, especially after the work of Peter Yodzis, who questioned the stability and vulnerability of sea animals in the ocean. In 1981, he found that the oceanic predator and prey relationship cannot be viewed as randomly connected as Robert May had assumed in his pioneering work on sea animal population mathematics in 1973 [45,46,47,48].

There is another possible bio-climate interaction due to the modulation of ocean circulation by the blooming of phytoplankton. This could be triggered by small-scale geophysical flows, i.e., internal gravity waves. It is known that internal gravity waves play a fundamental role in the ocean energy balance. Internal waves are efficient mixers of sea water. This enhances phytoplankton dynamics, which may impact the meridional oceanic heat flux, thus possibly causing the modulation of climate conditions.

Andrew Majda pointed out that one of the most challenging scientific topics of this century is to understand “turbulence in ocean and atmosphere”. He felt that this was fundamental if we are to ever understand the precise interactions of atmosphere, ocean, and land processes with fluid dynamical, biological, and chemical processes (p. 49) [49]. While his emphasis was on environmental diffusion problems, I would emphasize the need to develop a mathematical methodology for treating real-world diffusion processes. The challenges are enormous as this covers molecular and geophysical turbulence under the influence of rotating stratified geophysical fluid dynamics. Several tools are available to address this issue: non-Gaussian probability distributions for environmental and geophysical data, including distributions of rainfall amounts, and non-Gaussian probability distributions of diffusion, oceanic drifter path data, and ocean temperature time series data from ocean mooring stations. These methods should improve turbulent transport in the real-world stratified ocean [50,51,52,53].

## Data Availability

No database was used and no new data were generated for this article.

## Appendix A. Non-Equilibrium Thermodynamics Viewpoint

Non-equilibrium thermodynamics is a vast field of scientific endeavor with deep roots in physics and chemistry. It has applications in all branches of physical sciences and engineering and, more recently, in a number of interdisciplinary fields, including environmental research and, most notably, the biological sciences. The purpose of non-equilibrium thermodynamics is to describe transport processes with interactions between subsystems in a systematized way. It is especially useful in open system thermodynamics, as it describes transport processes such as the transport of electrolytes across inter-cell membranes. Recently, it has provided useful viewpoints in applications in biological processes.

Photosynthesis by oceanic phytoplankton is a manifestation of non-equilibrium thermodynamics as there are several simultaneous transport processes occurring. Two important examples are energy transport processes coupled with processes of the transport of electrons and matter and the transport of electric currents in phytoplankton and plants [19]. Moreover, communication of energy and matter with the environment is the basis of the non-equilibrium thermodynamic system for maintaining phytoplankton’s existence, rather than approaching a state of local equilibrium, the thermodynamic equivalent of death. Macroscopic changes in living beings are supported by a continuous inflow of low-entropy energy and low-entropy matter.

The fluid dynamics laboratory experiment that illustrates the “Benard convection process” is a beautiful example of a “dissipative structure” in the language of non-equilibrium thermodynamics. An application of non-equilibrium thermodynamic theory to the Benard convection example is a useful analog to living things. The fluid dynamic equations for Benard convection are basically evolution equations for energy and momentum. Benard convection is a simple example of the “application of non-equilibrium thermodynamics” as it requires a formalism of responsible estimates for energy and entropy flows. The application of energy and entropy formalism for photosynthesis is nontrivial as every chemical reaction must be accounted for. In contrast, the generalized process of “Life” can be expressed in terms of the language of quantum mechanics, i.e., electron excitation transfer processes to molecular complexes in the dark reactions of photosynthesis. Pigogine and Stengere demonstrated an approach for employing non-equilibrium thermodynamics in their treatment of the nonlinearity of chemical reactions that proved a branching of evolution, coined as the birth of a new state.

## Appendix B. Complexity System Science

Consider climate systems and living beings as two examples of complexity systems. Here, many subagents having different space and time scales are linked together through their interactions. They can also be thought of as forming parts of networks. For example, photosynthesis starts with the absorption of infrared light quanta by pigments at a time scale of 10^−15^ s, while the time scale of cell growth and multiplication is from 10^2^ to 10^3^ s. Photochemical reactions occur from 10^−9^ s to 10^−4^ s. Then, the whole time scale of biochemical reaction is from 10^−4^ s to 10^2^ s. Hence, biochemical processes and physiological processes overlap, and CO2 anabolism overlaps with constructive metabolism, which is connected to cell growth and multiplication. Consequently, interaction between population dynamics, along with phytoplankton masses and sea green plants’ may have ecological and climatic implications. Thus, the notion of networks is an integral part of complexity systems. In this context, it is interesting that, using years of observational data, Tsonis verified the climate system to be a scale-free-type network with increasing randomness after the mid-1970s [54]. This is a possible example of an emergent phenomenon typical of complexity systems.

Neil Johnson listed eight ingredients of complexity systems: Such a system contains a collection of many interacting objects or agents. The objects are affected by memory or feedback. The objects can adapt their strategies according to their history. The system is open. The system appears to be alive. The system exhibits emergent phenomena which are generally surprising and may be extreme. The emergent phenomena arise in the absence of an invisible hand or controller. The system shows a complicated mix of ordered and disordered behavior [6].

## Appendix C. Modeling Complexity Systems

Mathematical modeling of a system with many degrees of freedom is often practiced by choosing a small number of parameters presumed to control the system. However, these parameters may interact with an enormous number of intrinsic parameters beyond any parameters in the differential equations. By solving such “model” equations, human beings obtain answers to the “Model” question in the form of analytical functions or numerics. However, these are not answers to the real problem posed.

For example, some microscopic phenomena in the photosynthesis process, such as electron excitation transfer to molecular complexes in dark reactions of photosynthesis, are hidden and invisible to macroscopic observers. One instance is the catalytic process of photosynthesis, which does not appear in macroscopic models as the chemical reaction formalism for photosynthesis is in the form of a “Model”. This is what Carl F. Weiszacker pointed out as “*kritschen in Science*” and an “*Irreversiblitat*” embedded in the macroscopic picture of classical “*Physik*”.

## Appendix D. Navier–Stokes Equations and Local Equilibrium

The numerical ocean circulation models used in computational experiments consist of momentum equations for a fluid parcel, i.e., the Navier–Stokes equations, and diffusion equations for constituents, i.e., heat content and dissolved matter. The Newtonian principle in the momentum equation is time reversible without viscosity. But molecular and turbulent diffusion processes for heat and dissolved matter are time-irreversible. They are written in the formalism of a gradient transport paradigm that is not fully substantiated in geophysical flows (see the discussion of the gradient transport formalism in Appendix E).

The basis of local equilibrium is often attributed to Newtonian physics, which accounts for trajectories of point particles with zero volume and without external influences. This is inconceivable in the real world. Hence, I employ the terminology “a fluid parcel with a permeable boundary”. There appears to be no way to totally isolate a fluid system from its external environment. Some substances are very effective at preventing the flow of heat energy and matter, but no known substance or device appears to be capable of providing complete insulation. Nevertheless, the assumption of point particles without volume in fluid dynamics is widely accepted in both classical physics and quantum mechanics because of the long tradition of a Euclidean setting of absolute space and time.

Time symmetry in classical dynamics and quantum dynamics is identified with velocity reversal, which is only applicable to a single degree of freedom of the body of interest and not to the whole system. Here, it is worthwhile to recall that the three-body problem is not integrable for analytical solutions of the momentum equations. In such a system, final states do not depend on initial states. In general, thermodynamic equilibrium states are mere attractors of irreversible non-equilibrium states. This is why Priogine and Stengere employed, in their famous popular science book *Chaos and Order*, the terminology of “far-from equilibrium states” in biological and ecological applications.

The inconsistency of co-existence of a time-symmetric principle in Newtonian mechanics and the time asymmetry of irreversible turbulent diffusion in geophysical fluid dynamics leads to mathematical approximations generated by human choices of parameters. Usually, these do not come from fundamental principles of non-equilibrium thermodynamics with flows of energy and matter on fluid parcel boundaries.

In 1991, Carl F. Weiszacker stated that in the mathematical and computational formalism, the concept of existence in the real world is an approximation (“*ist der Objektbegrff selbst nur eine Approximation*”) in human epistemology (line 25–27, p. 96) [15]. It is worth asking a question of fundamental epistemology: “What is a model?” John Casti answered this question as follows: “Scientific model is a set of rules written commonly in mathematical languages such as well posed solvable differential equations or difference equations. Without observation, there is no place for science or any other reality generation mechanism to get a foothold” [55].

## Appendix E. Gradient Transport in the Diffusion Equation

In 1905, Albert Einstein introduced the concept of a “Thought experiment which mimics the process of molecular diffusion” by introducing a gradient transport formalism. Since then, nearly all instances of traditional turbulent transport in environmental problems are modeled by a more or less ad hoc analogy to Einstein’s gradient transport model (which is suitable for molecular diffusion).

Much later, at a symposium entitled “Turbulent diffusion in environment pollution”, in 1973, Stanley Corrsin gave a lecture where he proposed a novel approach for identifying some conditions which are necessary for the validity of a gradient transport model in a mean-free-path-type random transport process. He added that the partial success of gradient transport models in turbulence is largely fortuitous and certainly surprising [56] (p. 26). Nevertheless, papers introducing or using gradient transport models proliferate with no hint that they may be wrong in principle. Surprisingly, they sometimes yield good agreements with experiments, although this success may be attributed to the number of empirical constants available.

## Appendix F. Thought Experiment for Nonlinear Dynamics in Noise-Forced Non-Equilibrium State

Starting with a general form of an isomerization reaction, together with chemical reactions mimicking life and entropy production, Prigogine and Stengere arrived at a standard far-from-equilibrium phenomenon. This is the “co-existence of two stable stationary states”, giving birth to the phenomenon of “hysteresis”. As external noise fluctuates, the zone of co-existence of two stable states increases, and for certain values of parameters, the co-existence of multiple stable states is possible.

Hysteresis is a state that cannot be recovered from its initial conditions. This is a topic discussed in “Catastrophe Theory” in engineering applications and “bifurcation” in nonlinear differential equations. Notice that “bifurcation” occurs in a parameter for which multiple stable states exist. If a parameter increases sufficiently slowly, the state remains close to one stable state (which mathematicians may say is “trapped by an attractor”). If the attractor disappears, or some independent parameter overrides it, then the state may jump to another attractor and not jump back to the first attractor. This introduces the notion that attracting sets are “observable” states of dynamical systems. David Ruelle wrote, “this non-reversible behaviour is called “hysteresis”. It is one expression of the fact that the choice of attrator made by a system depends on the history of the system” [57] (p. 44).
